# Aerobic Training Improves Angiogenic Potential Independently of Vascular Endothelial Growth Factor Modifications in Postmenopausal Women

**DOI:** 10.3389/fendo.2017.00363

**Published:** 2017-12-21

**Authors:** Pascal Izzicupo, Maria A. D’Amico, Andrea Di Blasio, Giorgio Napolitano, Fabio Y. Nakamura, Angela Di Baldassarre, Barbara Ghinassi

**Affiliations:** ^1^Department of Medicine and Aging Science, University “G. d’Annunzio” of Chieti - Pescara, Chieti, Italy

**Keywords:** exercise, menopause, angiogenesis, adipokines, vascular endothelial growth factor, inflammation

## Abstract

**Purpose:**

The purpose of this study is to evaluate the effect of walking-training on the balance between pro- and antiangiogenic signals and on the angiogenic potential in postmenopausal women.

**Materials and methods:**

Thirty-four postmenopausal women (56.18 ± 4.24 years) participated in a 13 weeks program of walking-training. Anthropometric measures, vascular endothelial growth factor (VEGF), interleukin (IL)-1α, IL-1β, IL-2, IL-8, IL-10, IL-12p70, tumor necrosis factor-α (TNF-α), C-reactive protein, insulin, IGF-1, cortisol, dehydroepiandrosterone sulfate (DHEA-S), leptin, visfatin, resistin, and adiponectin were evaluated before and after training. Moreover, serum samples were tested for their ability to chemo—attract endothelial cells and to support the *in vitro* formation of capillary—like structures.

**Results:**

After training, the levels of IL-8, TNF-α, leptin, and resistin were significantly lower, levels of DHEA-S and adiponectin increased, serum angiogenic properties improved, whereas no changes in anthropometric parameters or VEGF were detected.

**Conclusion:**

Walking training reduces inflammatory status and leads to a significant improvement in serum angiogenic properties in the absence of modifications in body composition and VEGF level.

## Introduction

The decline of circulating levels of estrogen that characterizes the menopausal transition is associated with three closely related events that may lead to the onset and persistence of cardiovascular (CV) diseases: changes in body—fat distribution (1, 2), systemic low-grade inflammation (3), and endothelial dysfunction (4). Endocrinological changes seen in menopause also affect angiogenesis and estrogen reduction may impair the regenerative capacity of the CV system (5), thus contributing to the age-related microvascular rarefaction. Physical exercise is an effective non-pharmacological intervention for CV disease prevention. Indeed, habitual physical exercise is shown to strongly benefit health and longevity in humans likely due, at least in part, to its vasoprotective effects. The mechanisms of vasoprotection conferred by exercise are likely complex (6) but include a significant improvement of endothelial function, the induction of anti-inflammatory response and a positive influence on the number and/or function of endothelial progenitors cells. Postmenopausal women, however, tend to be less active (2, 7), and physical inactivity determines a persistent, sterile inflammation that is regarded to endothelial dysfunction and vascular alterations (8, 9). Moreover, physical inactivity is often associated with an excess of adipose tissue, which has been demonstrated to secrete increased amounts of the proinflammatory cytokines, including tumor necrosis factor-α (TNF-α), interleukin 1-β (IL-1β), and IL-6, thus contributing substantially to the chronic systemic inflammation. More recently, adipose tissue has been shown to be a source of additional molecules that operate at the interface between metabolism, immune responses and vascular health. In particular, leptin is an adipokine structurally similar to the IL-6 that modulates different physiological actions including food intake and angiogenesis sustaining, in parallel, a proinflammatory environment; similarly, resistin modulates several metabolic and inflammatory pathways and plays a role in atherogenesis, whereas on the contrary adiponectin plays an important role in maintenance of endothelial function, displaying anti-inflammatory, and antiatherogenic properties (10). The increase in fat mass is also associated with a relatively high level of vascular endothelial growth factor (VEGF) (11), a potent angiogenic molecule that was first described as an essential growth factor for vascular endothelial cells (12). VEGF is produced by many cell types, including endothelial, adipose and skeletal muscle cells (13, 14). Physical exercise induces an increment of VEGF that sustain the growth of new blood vessels in the trained muscles; on the other hand, the higher VEGF levels observed in obese subjects seems to be ineffective since adipose tissue appears hypoxic and undervascularized.

In the present study, we evaluated the effect of moderate-intensity walking training on angiogenic potential in postmenopausal women. Our hypothesis was that aerobic exercise might improve the angiogenic capacity by determining combined actions on hormones, cytokines, and adipokines.

## Materials and Methods

### Participants and Study Design

The study was approved by the Ethical Committee of Chieti-Pescara University. The anthropometrical and physiological characteristics of the 34 postmenopausal women (56.18 ± 4.24 years) enrolled in the study are reported in Table 1. The inclusion criteria were: age < 65 years; menses naturally ceased for at least 12 months, with plasma estradiol <20 pg/ml; body mass index (BMI) >18.5 and <40 kg/m^2^; no estrogen-replacement therapy; no history of orthopedic disease/dysfunction/injury that would impair walking. Further requirements were no participation in regular exercise programs for the past 6 months or in controlled diet programs in the past 2 years before enrollment. Participants provided written informed consent. Both medical examination and measurements were performed in the morning, in controlled temperature (21–23°C) and humidity (50%) (15). The basal screening (*T*_0_) consisted of anthropometric measurements and body composition analysis, a physical examination and a maximal aerobic-fitness test. A 12-h overnight fasting blood sample was drawn for the biohumoral determinations to characterize the metabolic, inflammatory and hormonal profiles of each menopausal woman. In particular, we measured: VEGF; a panel of proinflammatory cytokines, including IL-1α, IL-1β, IL-2, IL-8, IL-10, IL-12p70, TNF-α, and C-reactive protein (CRP); insulin and IGF-1 concentrations; dehydroepiandrosterone sulfate (DHEA-S); cortisol; and some selected adipokines (leptin, visfatin, resistin, and adiponectin). Furthermore, the sera of the postmenopausal women were tested for their ability to chemo-attract endothelial cells and to support the formation of capillary-like structures *in vitro*. The same tests were repeated for comparison after participation in the physical exercise program (*T*_1_). The study design did not include a control group since bioactive molecules such cytokines and adipokines are affected by exercise or dietary interventions while the absence of lifestyle modifications is associated with stable and relative high levels of inflammatory markers (16).

**Table 1:** Physiological and anthropometric characteristics of the samples.

	Mean ± SD
Age (years)	56.18 ± 4.24
BMI	27.05 ± 4.43
FM%	34.85 ± 6.34
WC (cm)	85.60 ± 10.51
W/H ratio	0.83 ± 0.06
HR (bpm)	66.71 ± 8.18
SBP (mmHg)	127.50 ± 16.39
DBP (mmHg)	80.00 ± 8.26
TC (mg/dl)	236.71 ± 46.15
HDL (mg/dl)	59.00 ± 14.58
LDL (mg/dl)	154.45 ± 39.75
Triglycerides (mg/dl)	114.03 ± 71.34

*BMI, body mass index; FM%, fat mass percent; WC, waist circumference; W/H, waist to hip ratio; HR, heart rate; SBP, systolic blood pressure; DBP, diastolic blood pressure; TC, total cholesterol; HDL, high-density lipoprotein cholesterol; LDL, low-density lipoprotein cholesterol*.

### Anthropometry and Body Composition

All anthropometric measurements were performed by a specialist [level 3 certification of the International Society for the Advancement of Kinanthropometry (ISAK)]. Participants wore light clothing, had fasted, and had abstained from alcohol consumption from 48 h before the test. Body weight, stature, waist, and hip circumferences (WC and HC, respectively) were performed according to ISAK guidelines (17) using a stadiometer with a balance—beam scale and an anthropometric tape (Seca 200, Seca, Hamburg, Germany). BMI was calculated as weight (kilograms) divided by the square of stature (meters), whereas WC to HC ratio was calculated by dividing WC by HC. Body composition was assessed by the electrical bioimpedance technique using a foot-to-foot 50 kHz frequency bioelectrical impedance scale (BC-420MA, Tanita, Tokyo, Japan). The test was performed after voiding, in an upright position, barefooted and without conducting garments.

### Walking Training Eligibility, Aerobic Fitness Assessment, and Physical Exercise Program

According to the American College of Cardiology/American Heart Association joint guidelines (15), walking training eligibility and aerobic fitness assessment of participants were assessed on the basis of the Astrand protocol through a graded maximal test on a cycle ergometer (SANA BIKE 150F, Ergosana GmbH, Bitz, Germany). During the test, heart rate and rhythm control were monitored by continuous electrocardiogram (AT-10 plus, SCHILLER, Baar, Switzerland), and blood pressure was measured throughout the test.

The intervention consisted of walking training 4 days/week for 13 weeks at moderate intensity. Exercise intensity was monitored on the basis of a 15-point rating scale of perceived exertion (RPE) (18). Participants were familiarized with the scale before and during the first week of the training. The intervention was divided in three mesocycles progressively more challenging: during the first and second months of training, participants walked at RPE level 11 for 40 and 50 min/day, respectively, whereas during the third month the intensity was raised to RPE level 12–13 for 50 min. The walking training sessions were supervised and the compliance, calculated as the percentage of completed sessions to total sessions, was 81.9 ± 18.2%.

### Determination of Cytokine, Adipokine, and Hormone Levels

Sera and plasma were obtained from 12-h fasting blood samples and stored at −80°C until analysis. Concentrations of plasma cytokines (IL-1α, IL-1β, IL-2, IL-8, IL-10, IL-12p70, and TNF-α) were measured by the SearchLight Human Cytokine Array 1 (Aushon Biosystems, Billerica, MA, USA), whereas serum cortisol, estradiol, leptin, resistin, adiponectin, CRP, IGF-1, and DHEA-S levels were determined by enzyme-linked immunosorbent assays (DRG International Inc., Mountainside, NJ, USA). All samples were analyzed in duplicate during the same assay session.

### Endothelial Cell Migration and Tube Formation Assays

The human umbilical vein endothelial cell (HUVEC) line and culture medium were purchased from Cell Applications (San Diego, CA, USA). The cells were cultured in DMEM/medium 199 (DMEM-199) 50:50 containing 20% fetal calf serum (FCS), 2 mM l-glutamine, 100 U/ml penicillin/streptomycin, 5 µg/ml fibroblast growth factor (Peprotech, Rocky Hill, NJ, USA) and 17.85 UI/ml heparin at 37°C and 5% CO_2_. Before performing the migration and tube formation assays, cells were starved (3% FCS in absence of fibroblast growth factor) for 5 h.

The *endothelial cell migration assay* was performed using the BD BioCoat Angiogenesis System: Endothelial Cell Migration kit assay (Corning, New York, NY, USA). Briefly, 4 × 10^5^/ml starved HUVECs were placed in the upper layer of the cell-permeable membrane, whereas a solution of DMEM-199 with 5% serum obtained from each woman before or after the intervention program (*T*_0_ and *T*_1_) was placed in the chamber below. VEGF (10 ng/ml) was used as the procedure control. After 22 h of incubation, cells that migrated through the membrane were stained with calcein AM (8 µg/ml, BD), a fluorescent dye that specifically labels alive cells, and observed under a microscope and then quantified using a FC500 flow cytometer (Beckman Coulter, Indianapolis, IN, USA). At least three measurements were performed for each experimental condition.

For the *tube formation assay*, HUVECs (4 × 10^5^ cells/ml) were cultured on BD Matrigel matrix in DMEM-199 with 5% serum obtained from each woman at *T*_0_ and *T*_1_. After 16 h, endothelial cells were labeled with calcein AM (8 µg/ml, BD) and observed with the DM IRB microscope (Leica) equipped with a Coolsnap video camera (Roper Scientific Photometrics, Tucson, AZ, USA). MetaMorph 6.1 Software (Universal Imaging Corp., Sunnyvale, CA, USA) was used to quantify the tube formation by measuring the width and the area covered by the capillary-like structures in 5× acquired images. In particular, for each sample the capillary width was calculated as the mean of five randomly selected fields (10 different measures per field), whereas the area was determined by thresholding the fluorescence of cells and then expressing as a percentage of the thresholded area (% of surface covered by the capillary structures); for this evaluation, 10 measurements were performed for each experimental condition.

### Statistical Analysis

Data were initially screened for normally with Shapiro–Wilk statistic. Pearson correlation coefficient was used to evaluate correlations between anthropometric and humoral parameters. Differences between *T*_0_ and *T*_1_ were tested by the paired-sample *t*-test. Data are presented as mean ± SD. The level of significance was set at *P* ≤ 0.05.

## Results

### Determination of Cytokine, Adipokine, and Hormone Levels before and after the Walking Training

We analyzed the VEGF level, a panel of proinflammatory cytokines, factors involved in glucose metabolism such as insulin and IGF-1, adrenal hormones (DHEA-S, cortisol), and some adipokines (leptin, visfatin, resistin, and adiponectin) (Table 2). All of these molecules affect inflammatory processes and influence the vascular health.

**Table 2:** Cytokine, hormone, and adipokine levels before (*T*_0_) and after (*T*_1_) the intervention program.

		*T*_0_	*T*_1_	*t*	*P*
Growth factor	VEGF (pg/ml)	40.09 ± 3.72	41.44 ± 6.77	−1.58	>0.05

Inflammatory cytokines	IL-1α (pg/ml)	8.98 ± 13.44	10.02 ± 14.34	−0.57	>0.05
	IL-1β (pg/ml)	2.52 ± 3.66	1.91 ± 2.51	1.60	>0.05
	IL-2 (pg/ml)	79.37 ± 46.81	72.78 ± 42.72	1.08	>0.05
	**IL-8 (pg/ml)**	**25.29 ± 39.93**	**19.29 ± 28.13^a^**	**2.24**	**0.032**
	IL-10 (pg/ml)	4.44 ± 4.19	3.79 ± 3.13	0.89	>0.05
	IL-12p70 (pg/ml)	8.23 ± 18.91	8.06 ± 11.96	0.06	>0.05
	**TNF-α (pg/ml)**	**42.60 ± 31.25**	**26.75 ± 24.73^a^**	**3.26**	**0.003**
	CRP (ng/ml)	2933.54 ± 5172.55	1516.06 ± 1656.92	1.57	>0.05

Hormones	Insulin (pg/ml)	11.75 ± 6.45	12.64 ± 8.24	−0.65	>0.05
	IGF-1 (pg/ml)	83.53 ± 65.75	98.49 ± 104.23	−0.79	>0.05
	**DHEA-S (pg/ml)**	**0.95 ± 0.62**	**1.13 ± 0.75^a^**	**−3.73**	**0.001**
	Cortisol (pg/ml)	126.48 ± 55.50	109.85 ± 46.09	1.83	>0.05
	Estradiol (pg/ml)	10.00 ± 4.19	10.41 ± 3.23	−0.79	>0.05

Adipokines	**Leptin (pg/ml)**	**57.34 ± 26.72**	**48.52 ± 26.60^a^**	**3.18**	**0.003**
	Visfatin (pg/ml)	7.32 ± 14.08	7.71 ± 7.39	−0.15	>0.05
	**Resistin (pg/ml)**	**3.07 ± 1.74**	**2.24 ± 2.03^a^**	**4.92**	**<0.001**
	**Adiponectin (pg/ml)**	**17.95 ± 11.50**	**25.75 ± 16.92^a^**	**−3.59**	**0.001**

*Data are expressed as mean ± SD. Significance was set at P < 0.05*.

*^a^Significantly different from T_0_*.

*VEGF, vascular endothelial growth factor; IL, interleukin; TNF-α, tumor necrosis factor alpha; CRP, C-reactive protein; IGF-1, insulin-like growth factor 1; DHEA-S, dehydroepiandrosterone sulfate*.

The walking training did not result in a significant change of body weight, anthropometric measures or lipid profiles, but it did alter some bio-humoral factors (Table 2). In particular, we detected a significant reduction of IL-8 and TNF-α. This alteration of the cytokine profile was accompanied by an increase in DHEA-S and by changes in adipokine concentrations, specifically a reduction in leptin and resistin and an increase in adiponectin. However, we did not detect a significant change in VEGF level.

**Figure 1:**
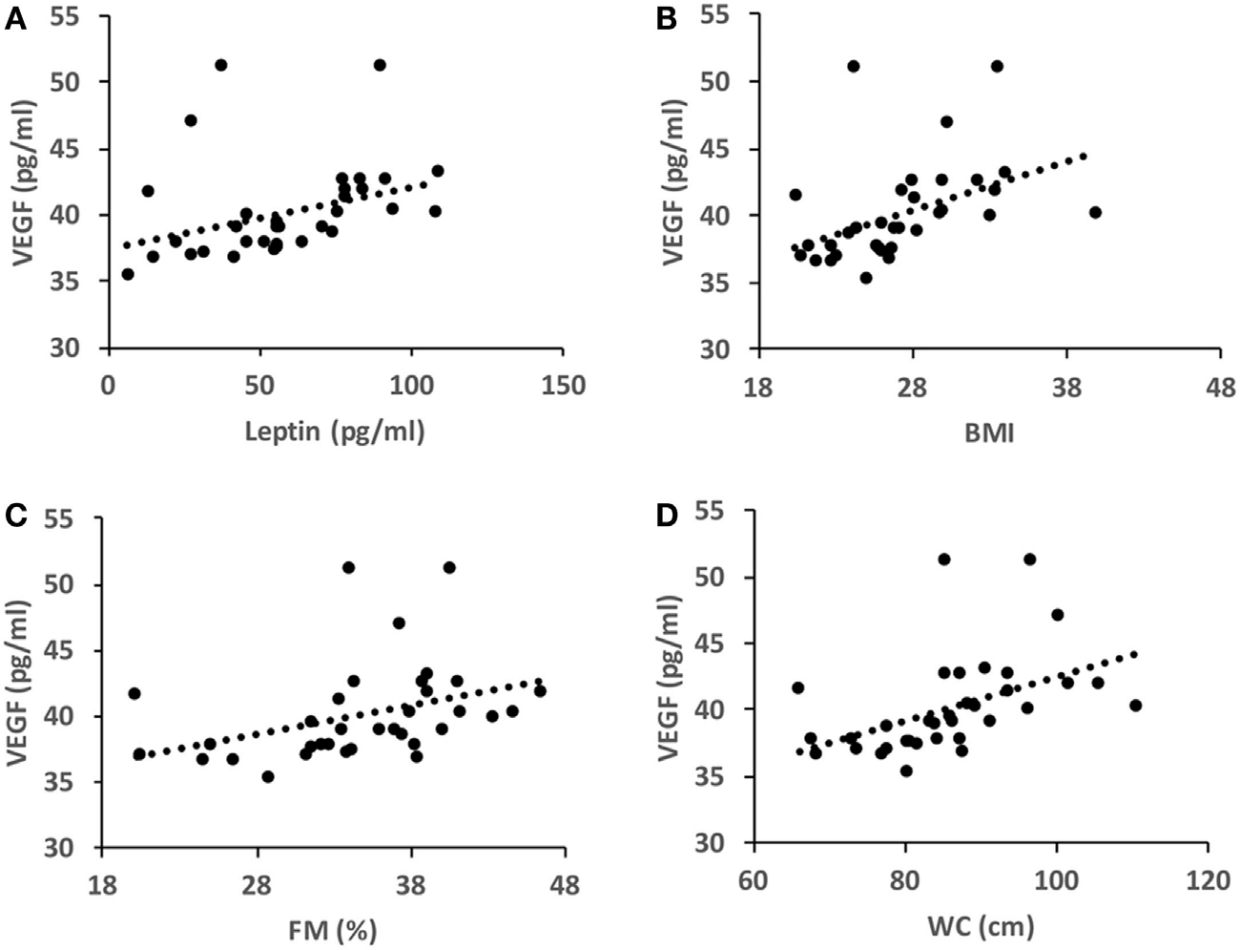
Pearson’s correlation coefficients in basal conditions (*T*_0_). **(A)** *r* = 0.343, *P* = 0.047; **(B)** *r* = 0.438, *P* = 0.01; **(C)** *r* = 0.468, *P* = 0.005; **(D)** *r* = 0.361, *P* = 0.036. BMI, body mass index; WC, waist circumference; FM%, fat mass percent.

Under basal conditions, VEGF level directly correlated with FM%, BMI, WC, and leptin level (Figure 1). These correlations were not found after the intervention program.

### Angiogenic Potential of Serum before and after Training

**Figure 2:**
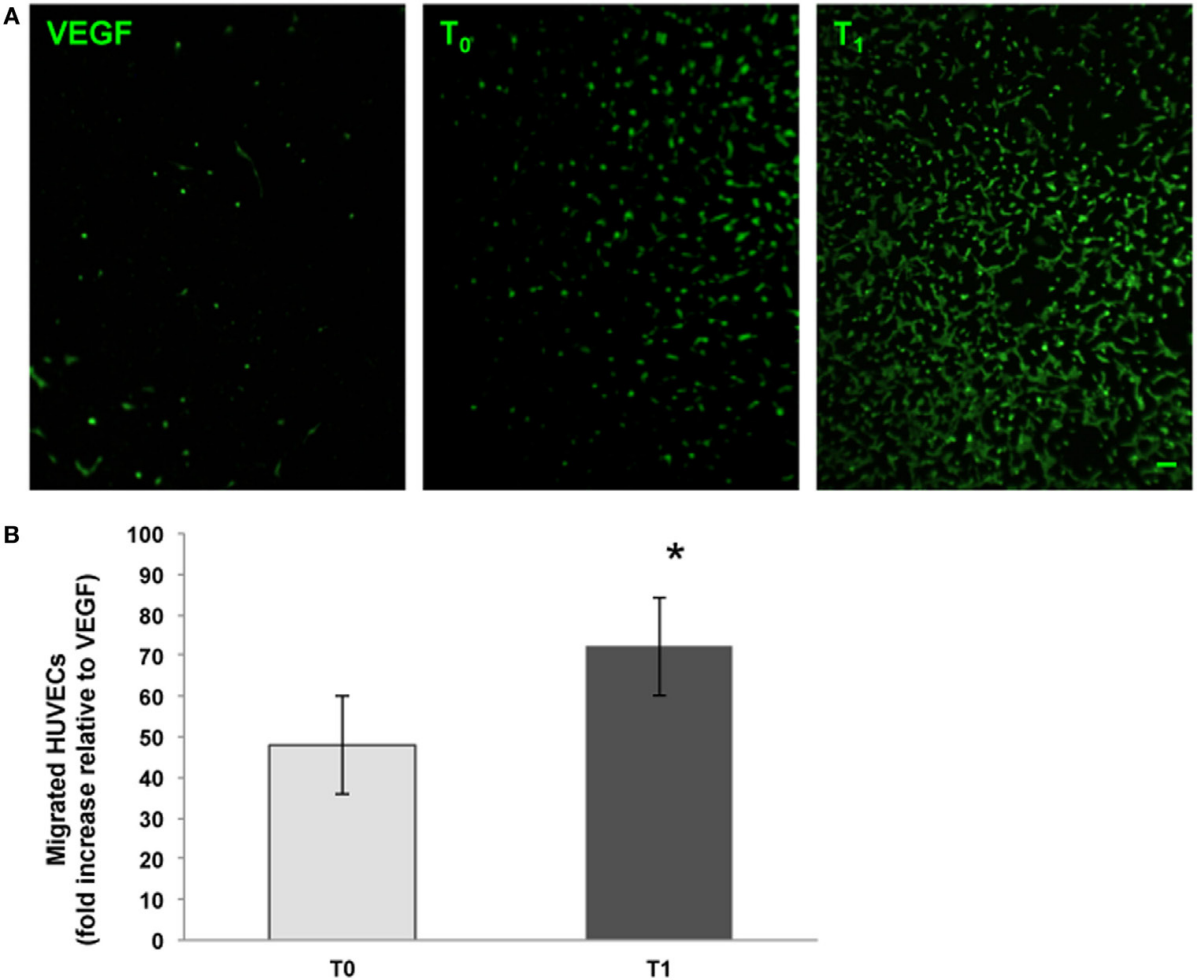
Endothelial cell migration assays. The chemoattractive potential of serum obtained from menopausal women before (*T*_0_) and after (*T*_1_) training was assessed. Vascular endothelial growth factor (VEGF) (10 ng/ml) was used as procedure control. Human umbilical vein endothelial cells (HUVECs) that had migrated were stained with the vital dye calcein AM (green fluorescence). Original magnification: 20×. Bar scale: 10 µm. The graph shows the number of migrated cells expressed as the fold increase relative to the control. *Significantly different from *T*_0_ (*t* = −10.84, *P* < 0.001).

**Figure 3:**
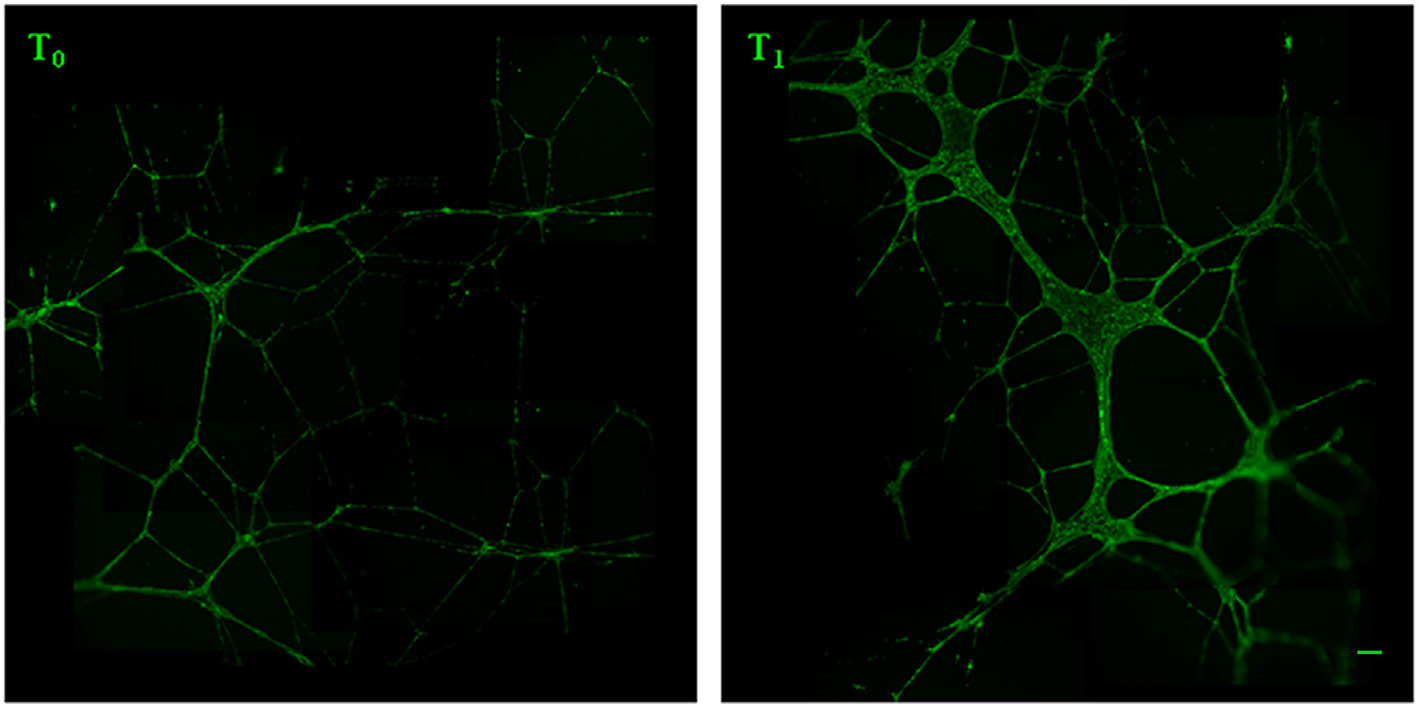
Analysis of capillary-like tube formation. Human umbilical vein endothelial cells were cultured in Matrigel with 5% serum obtained from the women before (*T*_0_) and after (*T*_1_) the training period. After 16 h, live endothelial cells were labeled with calcein AM (green fluorescence) and the tubes formation was analyzed by measuring the capillary-like structure width and area. Original magnification: 5×. Bar scale: 40 µm.

We analyzed the ability of the sera obtained before and after training to support angiogenesis by testing both the chemoattractive potential on endothelial cells and the ability to support the formation of capillary-like structures. It was found that walking training increased the chemoattractive potential of participants’ sera, as the number of cells that migrated through the membrane was significantly greater in the presence of posttraining sera (Figure 2, a fold change of 0.5 between T_0_ and T_1_ sera). Similar results were obtained by analyzing the *in vitro* development of capillary-like structures. As showed in Figure 3, both tube width and the area covered by capillary-like structures were significantly greater when endothelial cells were cultured in the presence of posttraining sera compared with pretraining sera (tube width: from 7.98 ± 7.83 to 18.25 ± 16.86 µm, *t* = −5.53, *P* < 0.001; area covered by tubes from 7.1 ± 4.8 to 16.99 ± 8.43%, *t* = −11.62, *P* < 0.001).

## Discussion

The main findings of this study are that: (i) in non-exercising postmenopausal women, VEGF concentration correlates with FM%, BMI, WC and leptin; (ii) walking training induces a significant improvement in serum angiogenic properties affecting the molecular network that connect the immune, hormonal, and adipose factors without alteration in VEGF, body weight, or body-fat composition.

The association between adiposity and VEGF has been widely investigated in recent years (14). As in previous studies (19, 20), our data show that VEGF is directly correlated with FM%, BMI, and WC. This observation is probably due to the fact that adipose tissue constitutively releases VEGF (21). VEGF sustains physiological angiogenesis during adipocyte expansion, whereas obesity is often characterized by hypoxic and undervascularized adipose tissue (22). This evidence suggests that VEGF efficacy is blunted in obese individuals whose adipose tissue is dysfunctional and inflammated (23). We also found for the first time to our knowledge, a direct correlation between VEGF and leptin. This finding is supported by the evidence that leptin stimulates VEGF synthesis *via* the JAK/STAT3 pathway (24). This observation contrasts with previous studies that did not find a correlation between VEGF and leptin; this discrepancy might be due to the fact that, in those other studies, the samples were heterogeneous for age and gender (18) or they were pathological samples (25).

After 13 weeks of walking training, we did not observe change in the weight or body fat distribution/composition of participants. This is unsurprising because walking training was the only experimental intervention, and compensation in both non-exercising physical activity and food intake could have prevented these effects (26). Nonetheless, physical exercise has several health benefits even in the absence of modification of anthropometric measures (27). Indeed, our data revealed an improvement in biohumoral factors, including significant reduction in the levels of TNF-α, IL-8, leptin, and resistin and an increase in adiponectin and DHEA-S Adipokines are endocrine factors that regulate metabolic functions and influence inflammatory responses. In particular, leptin and resistin promote the inflammatory and atherosclerotic process, whereas adiponectin has an anti-inflammatory function by modulating TNF-α (14). DHEA-S, a precursor for estrogen and androgen production, declines progressively with age. Even if this reduction is not gender related, it appears to promote greater risk in postmenopausal women for the concomitant decline in ovary function and sex hormone deprivation. Previous findings (28) have demonstrated that DHEA-S affects atherosclerosis by improving endothelial cell growth and survival and inhibiting TNF-α production, which is a primary factor in endothelial dysfunction. Our data confirm that aerobic training may improve vascular health by interfering with the cross-talk among the hormonal factors and the inflammatory processes (29–32).

Results on the VEGF response to exercise are heterogeneous. Several studies showed that exercise increases circulating levels of VEGF (33, 34), but some others showed no effects or decreasing levels (35, 36). Wahl et al. (37) suggested that the VEGF response is dependent on the exercise intensity: low-intensity exercise causes no changes or a decrease, while higher intensities cause an increase in circulating VEGF levels. Besides the influence of exercise intensity, the variation on circulating VEGF levels seems to depend also on the training status, with VEGF modification observed in endurance-trained subjects, but not in sedentary individuals (33). Our data evidencing in sedentary menopausal women no modification of plasma VEGF levels after aerobic exercise at moderate intensity are consistent with these observations.

Menopause is a risk factor for CV disease because the estrogen withdrawal has a detrimental effect on CV function and metabolism (38). Estrogen delays endothelial cell senescence and is virtually involved in every aspect of the angiogenesis process since it promotes endothelial progenitor cell migration and proliferative capacity and increases telomerase activity of endothelial cells (39). As consequence, the endocrinological changes of menopause induce vascular dysfunction also impairing the regenerative capacity of the CV system (5), thus contributing to the age-related microvascular rarefaction and determining a reduction of tissue perfusion and blood-tissue exchange (40, 41). These effects are worsened by the proinflammatory microenvironment of the vasal walls that causes endothelial dysfunction, affecting cell proliferation/survival and increasing microvascular cell apoptosis (42). In this study, we observed that walking training, despite not affecting VEGF level, improved the serum angiogenic capacity of postmenopausal women. This finding seem to suggest that physical exercise might counteract the menopause-related impairment of angiogenesis and microvascular rarefaction that contribute to a decline in peripheral blood flow and to a reduced perfusion at myocardial and cerebral levels (40). Because we found significant variation in cytokines, adipokines, and hormones but not VEGF modification, we hypothesize that the observed increased ability of serum to support angiogenesis after the intervention program may be ascribed, at least in part, to attenuation of the low-grade inflammation and hormonal and adipokine imbalances caused by the sex hormone deficiency.

## Conclusion

In menopausal women, physical exercise might determine reduction in inflammation and improvement in tissue perfusion that are important targets in the prevention of CV and other non-communicable chronic diseases. This goal can be easily achieved through regular walking training performed at moderate intensity, according to the international physical activity guidelines (43).

## Ethics Statement

All subjects gave written informed consent in accordance with the Declaration of Helsinki. The protocol was approved by the Ethics Committee of the Chieti-Pescara University.

## Author Contributions

PI designed the work and performed the experiments. MD designed the work and performed the experiments. ADBl performed the experiments. GN designed the work and revised the manuscript. FN analyzed the data and revised the manuscript. ADBa designed the work and wrote the manuscript. BG designed the work and wrote the manuscript.

## Conflict of Interest Statement

The authors declare that the research was conducted in the absence of any commercial or financial relationships that could be construed as a potential conflict of interest.
